# Biological and biophysics aspects of metformin-induced effects: cortex mitochondrial dysfunction and promotion of toxic amyloid pre-fibrillar aggregates

**DOI:** 10.18632/aging.101004

**Published:** 2016-07-28

**Authors:** Pasquale Picone, Silvia Vilasi, Fabio Librizzi, Marco Contardi, Domenico Nuzzo, Luca Caruana, Sara Baldassano, Antonella Amato, Flavia Mulè, Pier Luigi San Biagio, Daniela Giacomazza, Marta Di Carlo

**Affiliations:** ^1^ Istituto di Biomedicina e Immunologia Molecolare, CNR, Palermo, Italy; ^2^ Istituto di Biofisica, CNR, Palermo, Italy; ^3^ Departimento di Scienze e Tecnologie Biologiche, Chimiche e Farmaceutiche, University of Palermo, Palermo, Italy; ^4^ Current address: Italian Institute of Technology, Genova, Italy

**Keywords:** Alzheimer's disease, metformin, mitochondrial dysfunction, cell degeneration, mitochondrial pores, β-amyloid aggregation

## Abstract

The onset of Alzheimer disease (AD) is influenced by several risk factors comprising diabetes. Within this context, antidiabetic drugs, including metformin, are investigated for their effect on AD. We report that in the C57B6/J mice, metformin is delivered to the brain where activates AMP-activated kinase (AMPK), its molecular target. This drug affects the levels of β-secretase (BACE1) and β-amyloid precursor protein (APP), promoting processing and aggregation of β-amyloid (Aβ), mainly in the cortex region. Moreover, metformin induces mitochondrial dysfunction and cell death by affecting the level and conformation of Translocase of the Outer Membrane 40 (TOM40), voltage-dependent anion-selective channels 1 (VDAC1) and hexokinase I (HKI), proteins involved in mitochondrial transport of molecules, including Aβ. By using biophysical techniques we found that metformin is able to directly interact with Aβ influencing its aggregation kinetics and features. These findings indicate that metformin induces different adverse effects, leading to an overall increase of the risk of AD onset.

## INTRODUCTION

According to Rotterdam Study, individuals with metabolic pathologies such as Type2 diabetes (T2DM) or Obesity have almost a two-fold greatest risk of developing Alzheimer's disease (AD) [1]. AD is the most common cause of dementia in the elderly and it is associated with a progressive impairment of cognitive function, orientation, and difficulties with problem-solving or language. Thus, in patients with AD, gradually, over the time, more parts of the brain are damaged developing progressive symptoms, leading to death. Thirty-five millions of persons in the world are now considered to be affected by AD and this number is expected to double in the next few decades [2]. Even if the etiological defects in AD are not well known, prevalent ideas implicate build-up of soluble β-amyloid (Aβ) oligomers or insoluble plaques or neurofibrillary tangles [3,4]. Aβ is a 39–43 amino acid peptide formed from the cleavage of amyloid precursor protein (APP), a transmembrane glycoprotein. Neurofibrillary tangles are, instead, produced by hyperphosphorylation of Tau, a protein associated with microtubules in neurons [5]. Aging is the primary risk factor for the development of AD and many other pathological conditions occurring in older people, including T2DM. Change in cognitive function and increase of neurodegeneration markers were found both in patients with T2DM and/or obesity [6] and in insulin-resistant obese mice [7], suggesting the existence of a common molecular mechanism. Some studies have identified in insulin resistance condition the link among the pathophysiology of metabolic disorders and the brain alteration [8–10]. Insulin has a significant role in modulation of synaptic plasticity and learning memory and a high number of Insulin Receptor (IR) are present in brain [9,11]. Modification in the insulin concentration and IR number have been reported in AD cell model [12] and AD brain, leading to the result that AD can be considered as a brain diabetes or “Type 3 Diabetes” [13]. On the basis of this association between metabolic disorders and impaired cognition it should be relevant to investigate whether a potential risk or benefit could occur by using antidiabetic treatments on brain health [14,15].

Evidences on the association between antidiabetic medication and the risk of AD are conflicting and not well documented [16]. One of the few classes of therapeutics, efficient in lowering glucose production are the biguanides, which include molecules as phenformin (2-N-phenethylcarbamimidoyl guanidine) and metformin (1,2-dimethylbiguanide hydrochloride). In particular, the last is the most frequently prescribed drug for T2DM or other metabolic diseases. On the basis of its physicochemical structure and properties, metformin is a small amphoteric molecule (129 Da) with pKa values of 2.8 and 11.5. These characteristics are associated to high water solubility and low lipid solubility. Studies both in vitro and in vivo, indicate that metformin increases the production of Aβ [17], suggesting that its long-term administration may promote AD onset. On the contrary, a neuropathological study has reported that people treated both with insulin and oral antidiabetic drugs had developed a significantly lower amyloid plaque density [18]. A recent population-based case-control study examined the relationship between T2DM and administration of different antidiabetic drugs and risk of AD development. The authors conclude that long-term users of metformin may have a somewhat higher risk of AD onset and development [19]. However, poor information is available about the molecular mechanism activated by metformin. Some reports indicate that it is able to stimulate AMP activated protein kinase (AMPK), an enzyme activated when cellular energy levels are altered [20]. Recently, in vitro and ex vivo studies have demonstrated that metformin favors APP and presenilin increase and induces Aβ production and aggregation [21]. Furthermore, metformin acts as a pro-oxidant molecule inducing oxidative stress and mitochondrial dysfunction that, in turn, activates Nf-κB, a transcription factor involved in regulation of APP and presenilin gene expression. Lastly, these molecular mechanisms are counteracted by insulin co-administration [21].

Functional and structural mitochondrial defects contribute to the pathogenesis of aged-related diseases. Metformin affects mitochondria by inducing depolarization of the mitochondrial phospholipidic membrane [21,22] and inhibiting the mitochondrial complex I of the respiratory chain [23,24]. Moreover, the use of metformin changes the expression of several proteins involved in metabolic processes, the regulation of apoptosis and the structural preservation of brain mitochondria [25]. Impairing of exchange of molecule between cytoplasm and mitochondria is one of the cause of mitochondrial dysfunction. The Translocase of the Outer Membrane (TOM) complex, of which TOM40 is the key subunit, is the main gateway for the import of most mitochondrial proteins synthesized in the cytoplasm. The complex is relevant also for mitochondrial biogenesis and its damage triggers mitochondrial dysfunction [26]. Furthermore, opening and closure of the mitochondrial permeability transition (MPT) pore, in which voltage-dependent anion-selective channels 1 (VDAC1), also known as mitochondrial porin, is one of the main proteins, is impaired in patients with neurodegenerative diseases [27]. Moreover, VADC1 interacts with hexokinase I (HKI) and this binding protects against cell death [28]. Thus, the correct mitochondrial transport of ions, metabolites and molecules affects cell survival and death mechanisms.

From a molecular point of view, the overproduction and aberrant self-assembly of the amyloid β peptide (Aβ) into fibrillar aggregates constitute the first step of the so-called amyloid cascade hypothesis, thought to trigger AD [29]. These extremely toxic oligomers [30,31] have high hydrophobicity, are small [32] and constitute a heterogeneous group characterized by several highly dynamic different assemblies with multiple conformational states. Although the mechanism of cytotoxicity is not yet fully understood, it has been ascertained that amyloid oligomers are the most toxic species [30,31]; in fact, they directly interact with and affect cell plasma membranes by forming pores and consequently disrupting several cellular processes. Amyloid fibrils have also been recently demonstrated to modify the membrane integrity. In fact, they interacting with lipid bilayers are destabilized and disassembled in the pre-fibrillar toxic forms, inducing cell dysfunction, although to a lesser extent [31,33–35].

From a molecular point of view, the self-assembly of Aβ peptides in well-ordered fibrils constituting the senile plaques found in AD brains, is a complex process composed by several steps. It is characterized by multiple transitional aggregation species as initial seeds, soluble small oligomers, protofibrils and insoluble amyloid fibrils, with a β-sheet conformation. The kinetics of amyloid formation is best described by a sigmoid curve and can be schematically described in three stages [36,37]:

1. the slow lag nucleation phase, in which monomers gradually undergo a secondary structure conformational change from random coil to β-sheet and associate to form oligomeric nuclei/protofibrils;
2. the fast exponential elongation phase, in which the soluble species are progressively arranged at the ends of preformed β-sheet rich structures in a thermo-dynamically favorable process. The initial oligomeric nuclei rapidly grow by further addition of monomers forming larger fibrils;
3. the saturation phase, in which the fibrils are completely formed and associate each other giving rise to stable mature fibers.

In this study we assessed the molecular effects of metformin in specific brain area of mice. Difference in

Aβ deposits, expression of AD markers and proteins involved in mitochondrial dysfunction were found between cortex and hippocampus regions of quite young mice. Furthermore, the direct interaction between metformin and Aβ aggregate formation was determined by in vitro biophysical study.

## RESULTS

### Metformin is a fluorescent molecule able to reach the brain

**Figure 1:**
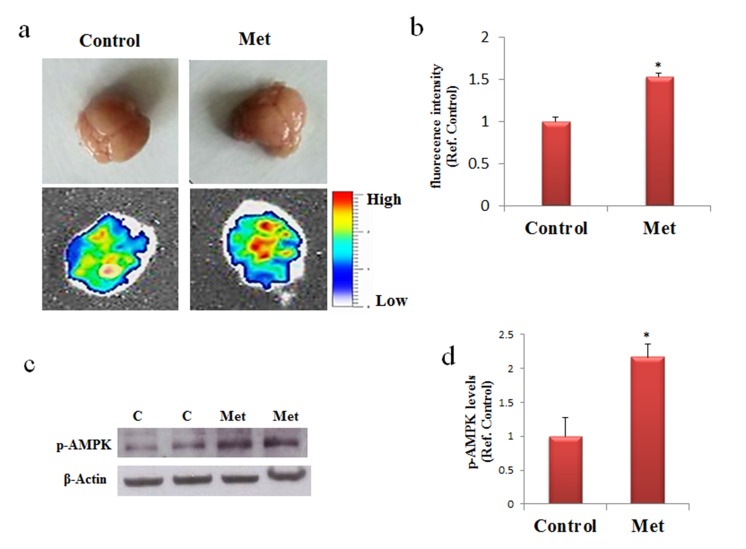
Metformin reaches the brain and activate AMPK **(a)** and **(b)** Mice were untreated or treated with metformin for seven days and the fluorescence in the brain was quantified by bioimaging. Fluorescence values are referred to the control. **(c)** Western blot of proteins extracted from brains of metformin untreated (C) or treated mice (Met) and incubated with phospho-AMPK (p-AMPK) and β-Actin (loading control) antibodies. **(d)** Quantification of immunoreactivity using densitometric analysis. Representative images from 2 animals for condition are shown. n=5 per group.

Absorption or fluorescence phenomena of ultraviolet or visible lights by a molecule depend on electron transitions between molecular orbital energy levels. Due to the presence of two double conjugated bonds the possibility of fluorescence phenomenon in metformin has been investigated. The emission spectrum obtained by fluorescence measurements indicates that metformin, once excited at 395 or 488 nm, has the emission peaks at 475 and 520 nm, respectively (data not shown). This property allows analyzing the presence of metformin into the brain of mice after its dispersion in drinking water. Metformin was administered to C57B6/J mice and after seven days the mice were sacrificed and the brains analyzed by using an imaging instrument. A strong signal was detected in the treated mice whereas no signal was found in the control, thus indicating that metformin has reached the brain (Fig. 1a,b). The delivery of metformin to the brain was confirmed by the increased levels of phosphorylated AMP-activated kinase (AMPK), one of the main molecular target of metformin (Fig. 1c,d). Thus, metformin crosses the blood brain barrier (BBB) and has an impact on the brain biochemical pathways.

### Chronic metformin treatment stimulates APP processing mainly in brain cortex region

**Figure 2:**
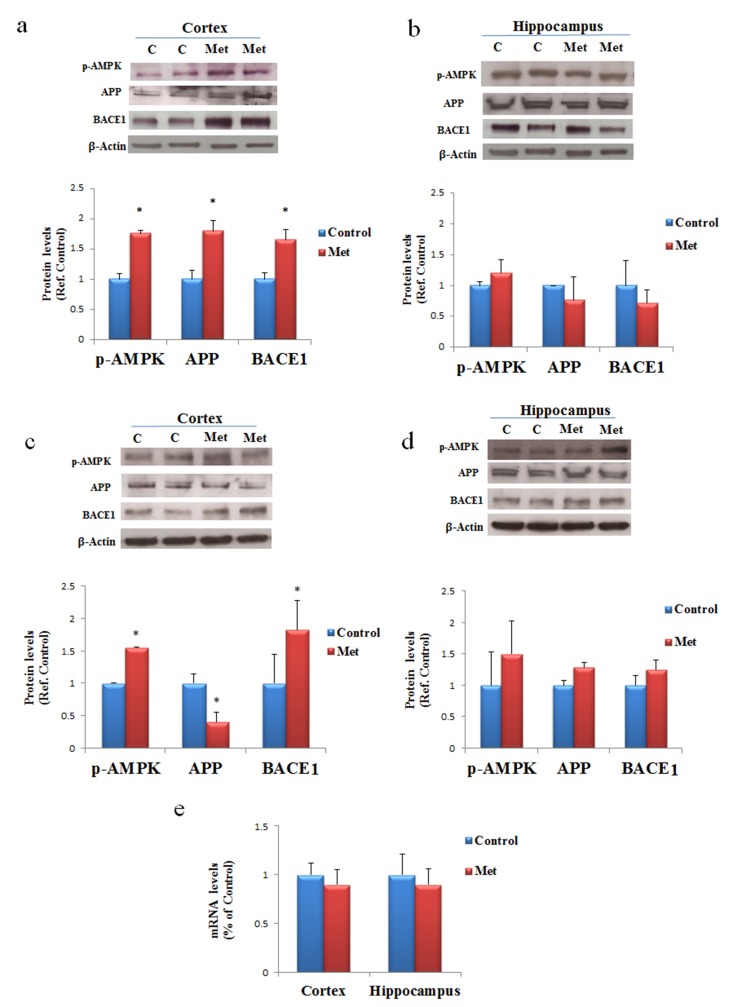
AD biomarkers are differently activated by metformin treatment in brain regions Western blot of protein extracted from brain lysates of mice cortex **(a)** and hippocampus **(b)** after seven days of metformin treatment and incubated with anti-phospho-AMPK (p-AMPK), APP, BACE and β-Actin (loading control) antibodies. Western blot of protein extracted from brain lysates of mice cortex **(c)** and hippocampus **(d)** after chronic metformin treatment and incubated with anti-phospho-AMPK (p-AMPK), APP, BACE1 and β-Actin (loading control) antibodies. Quantification of immunoreactivity was performed using densitometric analysis. **(e)** Effect of chronic metformin treatment on APP transcript levels determined by quantitative real-time PCR in cortex and hippocampus regions. n=5 per group.

To ascertain the possibility that metformin is a risk factor for AD onset, especially in long term administration, C57B6/J mice were treated with metformin for seven days or three months. After these treatments proteins extracted from cortex and hippocampus, two-brain area mainly damaged in AD, were submitted to Western blot. Changes in the levels of BACE1, an enzyme required for APP processing to produce Aβ, its pathogenic cleavage product, and the same APP were measured. Activation of AMPK was evaluated for confirming the biochemical activity of metformin in the brain (Fig. 2). An increase of the levels of BACE1 and APP expression was detected in the cortex after seven days of treatment (Fig. 2a). In contrast, an increase of BACE1, and a decrease of APP were found after chronic treatment in the cortex, suggesting that an enhanced processing may be occurred (Fig. 2c). Furthermore, after both treatments, no significant differences in BACE1 and APP expression in the hippocampus were detected (Fig. 2b, d). To validate the hypothesis that the decreased presence of APP in the cortex was a consequence of the β-secretase increased activity, a quantitative real-time PCR (qRT-PCR) experiment was performed. No significant change was observed in APP transcript, strongly signifying that the produced protein was quickly processed (Fig. 2e).

### Metformin induces accumulation of Aβ aggregates mainly in brain cortex region

**Figure 3:**
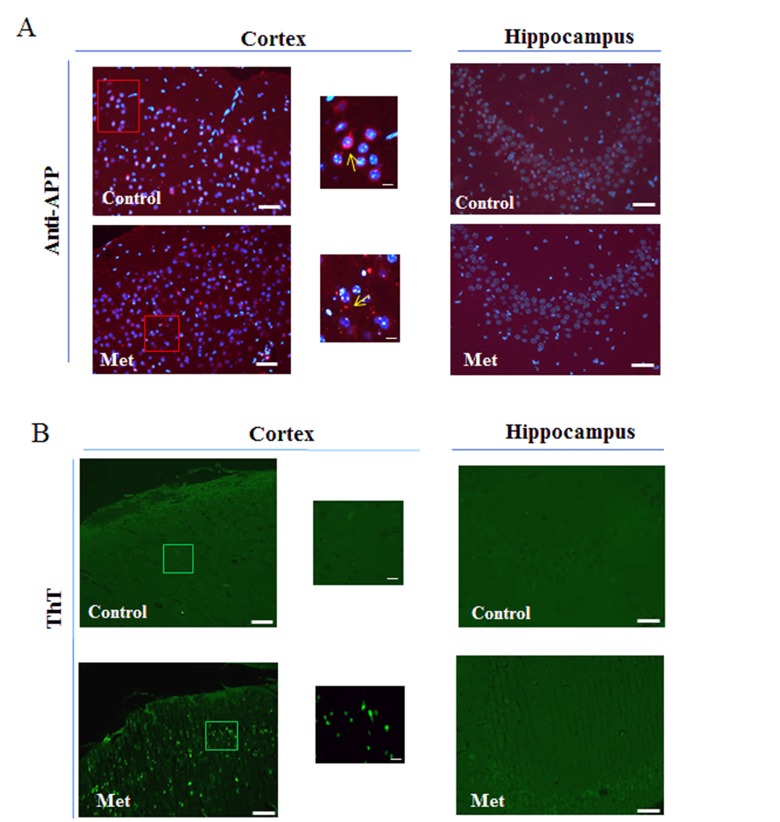
Metformin induces accumulation of Aβ aggregates **(a)** Immunofluorescence of cerebral cortex and hippocampus sections of metformin treated mice stained using anti-APP. Nuclei were stained with Hoechst 33258 and merged images with anti-APP staining are shown. **(b)** ThT staining of Aβ aggregates on coronary sections. High magnification of the squared areas is shown. Yellow arrows in the zoomed images indicate diffuse or punctate anti-APP staining. 20X original magnification. Scale bars = 50 μm and 5 μm in the zoomed images. n=5 per group.

Since the obtained results after three months of treatment suggested an augmented processing of APP mainly in the cortex, we explored the possibility that an increase of Aβ production could have enhanced its aggregation and deposition in the extracellular area. By immunofluorescence analysis, using coronary brain sections, in which cerebral cortex and hippocampus were visible, and anti-APP antibody we observed a diffuse staining around the nuclei of the control and a punctate staining around the cells of treated cortex, suggesting presence of aggregates due to increased processing (Fig. 3a). However, this result was confirmed by staining with Thioflavin T (Fig. 3b), a dye used to reveal the presence of β-sheet protein aggregates because of the increase of its fluorescence emission intensity upon binding to the linear array of β-strand aggregates [38,39]. In particular, we observed aggregates with a dimension ranging below 1 μm mainly in the cortex (Fig. 3b). In contrast, no significant immunoreactivity, or presence of Aβ aggregates, was detectable in hippocampus (Fig. 3a, b).

### Metformin induces mitochondrial dysfunction by impairing MPT pores and membrane channels

**Figure 4:**
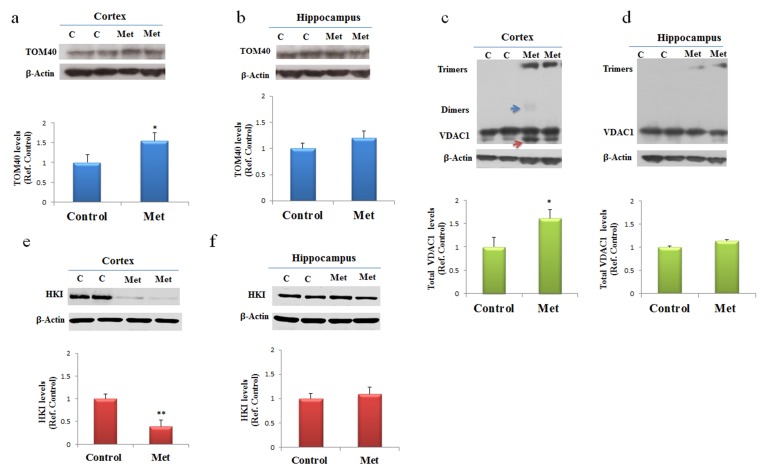
Metformin after chronic administration, changes TOM40 expression levels and induces VDAC1 oligomerization, and affects Hexokinase I (HKI) in the cortex region Western blot of protein extracted from brain lysates of mice cortex **(a, c, e)** and hippocampus **(b, d, f)** after chronic metformin treatment and incubated with anti- TOM40, VDAC1, Hexokinase I (HKI), and β-Actin (loading control) antibodies. Quantitative analysis of total VDAC1, HKI and TOM40 levels relative to β-actin was performed using densitometric analysis. The blue arrow indicates a VDAC1 dimers and the red arrow indicates VDAC1 monomers with modified electrophoretic mobility. n=5 per group.

Specific mitochondrial proteins involved in distinctive structures regulate cross talk and transport of proteins and metabolites between cytoplasm and mitochondria. We addressed the question if metformin treatment might contribute to the alteration of the components of mitochondrial machinery. After three months of treatment changes in levels of expression were analyzed for some proteins involved in mitochondrial transport of different molecules and markers of mitochondrial dysfunction such as TOM40, VDAC1 and HKI. Western blot of proteins extracted from both cortex and hippocampus of mice treated with metformin and controls were incubated with antibodies against TOM40, VDAC1 and HKI. In agreement with previous data, Western blot analysis showed changes in the levels of expression of the analyzed proteins only in the cortex region. An increase of TOM40 was detected indicating an impairing of MPT pores (Fig. 4a). Further, an increase of total VDAC1 levels were observed in cortex where, in particular, we found together with VDAC1 monomers some dimers and trimers, suggesting that a fraction of the VDAC1 in the membrane is organized in oligomers (Fig. 4c). Moreover, the lower band, indicated by the red arrow in Figure 4C, represents a monomeric species whose electrophoretic mobility is modified by the intramolecular crosslinking of the VDAC1 N-terminal domain. A decrease of HKI amount was also detected suggesting that probably a detachment from VDAC1 was occurred (Fig. 4e). TOM40, VDAC1 and HKI levels were not significantly affected in the hippocampus brain area (Fig. 4b, d, f).

### Metformin induces neuronal apoptosis

**Figure 5:**
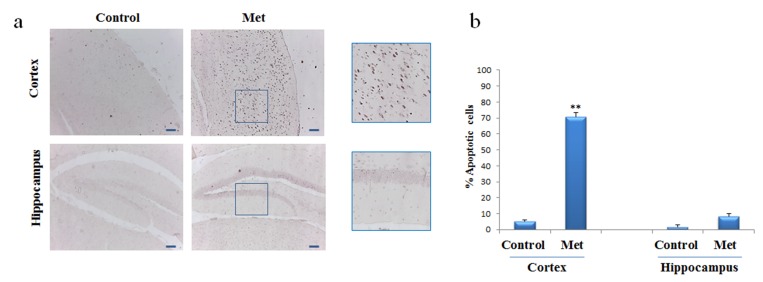
Metformin induces apoptosis mainly in neurons of cortex region TUNEL assay was performed on the paraffin sections of cortex and hippocampus obtained from mice untreated or treated with metformin to detect apoptotic nuclei (brown). **(a)** Representative cortex and hippocampus brain sections stained with TUNEL in control and metformin treated mice. High magnification of the squared areas is shown. **(b)** The histogram indicates the percentage of positive cells both in cortex and hippocampus regions normalized to the control. 10X original magnification. Scale bars = 100 μm. n=5 per group.

To assess whether, after chronic metformin treatment, apoptotic cell death occurred in vivo, presence of fragmented DNA was examined in cortex and hippo-campus brain sections by the terminal deoxynucleotidyl transferase-mediated, dUTP, nick end labeling (TUNEL) method. TUNEL-positive cells in brain sections of metformin treated mice were markedly higher in cortical region than in hippocampal region or control (Fig. 5a, b).

### Metformin directly interacts with Aβ peptide influencing its aggregation kinetics in vitro

**Figure 6:**
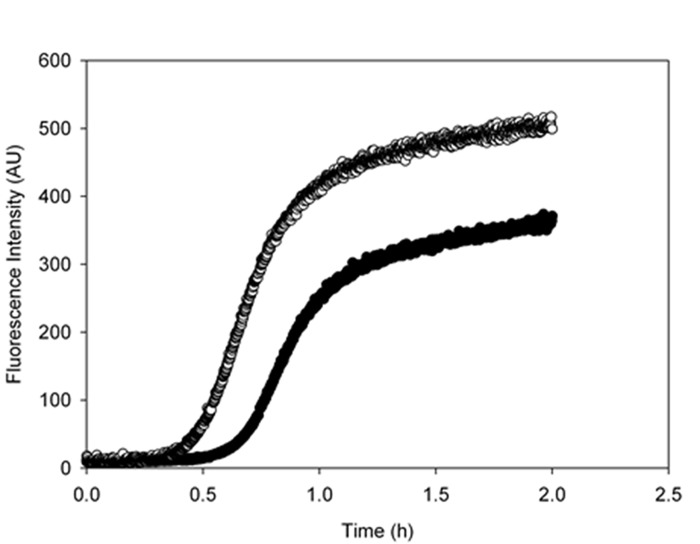
Metformin increases the lag-phase duration and reduces the final amount of amyloid fibrils Aggregation kinetics of 50 μM Aβ_1-40_ was followed in the absence (empty circles) and in the presence (black circles) of 2mM metformin by ThT assay. The dye concentration was 12μM.

In order to assess whether metformin is also able to directly interact with Aβ peptide influencing its aggregation process we performed the fibrillogenesis kinetics by ThT assay. Figure 6 shows the time course of the ThT signal during the fibrillation kinetics of 50 μM Aβ incubated at 37°C in the absence and in the presence of 2mM metformin. The kinetics followed the typical nucleation-polymerization process, described by the sigmoidal profile [36]. In the presence of metformin, the lag phase increased and the amount of final fibrils is reduced in comparison with control. Metformin addition seemed to reduce the effective Aβ concentration leading to initial nuclei, thus interfering with lag-phase.

**Figure 7:**
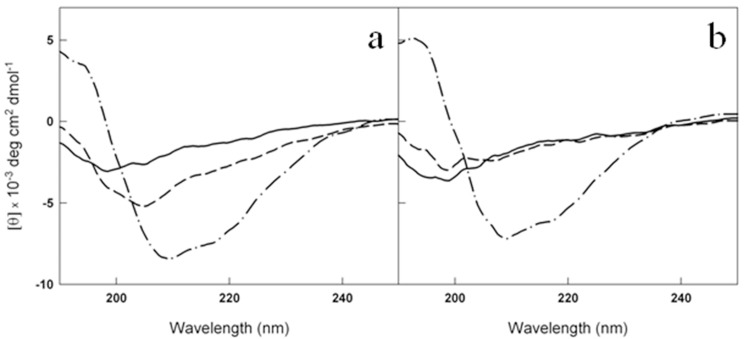
Metformin delays the conversion of Aβ_1-40_ from disordered coil to β-sheet structure Far-UV CD spectra of 50 μM Aβ_1-40_ at t0 (solid lines), t=0.5 h (dashed lines) and t=2h (dot dashed lines) at 37°C in the absence **(a)** and in the presence **(b)** of 2mM metformin. The signals from the buffer and metformin in buffer have been subtracted from the spectra in **(a)** and **(b)**, respectively.

This result was confirmed by circular dichroism (CD) experiments. CD spectra are sensitive to the secondary structure variation accompanying amyloid cross-β-structure formation during fibrillogenesis. In the initial phase of the aggregation process, the Aβ CD spectrum presents a single minimum that, typical of a random coil structure, is around 200 nm. The minimum shifts towards higher wavelength during amyloid formation. Our results have shown that the presence of 2mM of metformin delayed the β-sheet structure formation of a 50 μM Aβ1-40 solution. In fact, while the control sample reached the end point of the aggregation reaction (Fig. 7a) after 0.5 h, no significant changes were observed in the presence of the drug in the same time interval indicating that the peptide retained its disordered structure (Fig. 7b), in fully agreement with the fluorescence data. Always in accordance with ThT assay, after 2 h both the samples completed their conversion.

**Figure 8:**
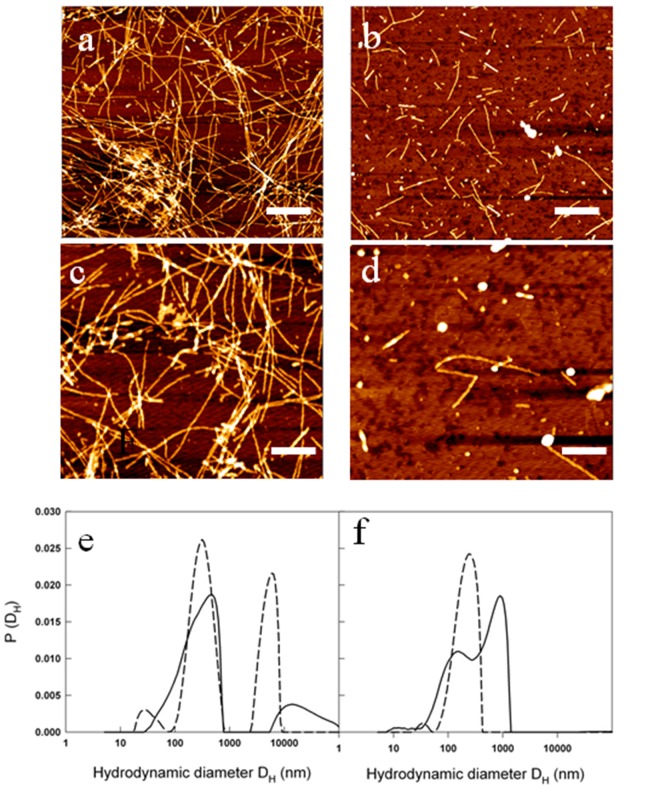
Metformin effects on fibril size and growth **(a-d)** AFM images acquired for: 50 μM Aβ_1-40_ at the end of the kinetics at 37°C and 200 rpm at two different magnifications: scale bar = 1μm and Z-range = 8.9 nm **(a)**; scale bar = 500 nm; Z-range = 8.3 nm **(b)**. The samples were compared with 50 μM Aβ_1-40_ + 2 mM metformin at the end of the kinetics at 37°C and 200 rpm at two different magnifications: scale bar = 1μm and Z-range = 7.0 nm **(c)**; scale bar = 500 nm; Z-range = 8.3 nm **(d)**. Particle size distribution from DLS of 50 μM Aβ_1-40_ incubated at 37°C and 200 rpm in the absence **(e)** and in the presence **(f)** of 2mM metformin after 0.5 h (dashed lines) and 2 hrs (solid lines) from the beginning of amyloid aggregation process.

The reduced amount of Aβ1-40 fibrils detected by ThT assay in the presence of metformin was confirmed by AFM measurements. In Figure 8a-d the morphology of the aggregates formed at the end of the kinetics in the absence and in the presence of metformin are reported. Amyloid fibrils result reduced in number and length when metformin is added in solution. Moreover, prefibrillar oligomeric aggregates are observed at the end of the kinetics of the peptide incubated with the drug.

The characterization of the action of metformin on the aggregation process of Aβ1-40 peptide was followed by Dynamic Light Scattering (DLS) experiments. Although not specifically focused on chemico-physical features characterizing amyloid formation, like conversion to cross-β-structure, the addition of DLS to other biophysical techniques provides important quantitative information on the hydrodynamic size variation occurring during an aggregation process. In this sense, it represents a suitable technique to monitor the influence of an exogenous molecule on the protein assembly, and, therefore, it is often used for testing drugs for therapeutic purposes. Moreover, light scattering technique does not need the use of extrinsic probes, whose evaluation of their potential influence on the process under study requires high carefulness [40]. Figures 8e and f show the hydrodynamic diameter DH distribution functions of a sample of Aβ1-40 peptide undergone to the amyloid formation protocol (37°C and 200 rpm under stirring) with and without metformin, respectively after 0.5 h from the beginning of the process and at the end of the experiment observation (2 h). The increase in ThT assay (Fig. 6) and variation in CD spectra (Fig. 7) reveal that, at that time, in the absence of metformin a significant amyloid cross-β-structure has already formed. Correspondently, two size distributions are revealed by DLS analysis: one centered at around 300 nm and the other one corresponding to larger species of the order of microns. At the same time, in the presence of metformin this higher size species is not formed and only the appearance of species at around 300 nm occurs.

The size distribution for samples collected at the end of aggregation kinetics reveals for the Aβ1-40 sample grown in the absence of metformin, a marked shift towards higher values in comparison to the initial time, probably corresponding to mature fibril formation. In contrast, only a little increase in the average hydrodynamic diameter (from 300 to 600 nm) in the correspondent sample incubated with the drug is detectable. The overall results from biophysical techniques converge in indicating that the metformin delays amyloid aggregation of Aβ1-40 peptide, reduces the amount of mature fibrils formed and, more important, stabilizes prefibrillar oligomeric species.

## DISCUSSION

We analyzed both biological and biophysical aspects to understand the molecular mechanisms induced by metformin and leading to neurodegeneration in mouse brain. Fluorescence measurements of ex vivo mouse brain performed by using the intrinsic fluorescence signal of the metformin gave us the direct confirmation that the drug had reached the central organ by crossing the BBB. For mouse treatment, an experimental dose, deduced by those utilized in other works, which uses the same C57B6/J strain, was employed [17,21]. In particular, Chen et al. administrating 2mg/ml, and using liquid chromatography-mass spectrometry, measured that metformin reaches a concentration of about 2 μM and 1 μM in the plasma and in the brain, respectively, well below the 10–40 μM achieved in human plasma [20]. Moreover, since the drug is eliminated via renal secretion in few hours, the mice were treated for three months to determine the incidence of long-term administration on the risk of AD developing. Activation of AMPK was considered as a marker of metformin-induced biochemical answer in brain, even if we cannot exclude that the response to metformin could not be limited only to its activation, but mediated by additional mechanisms depending directly or not by AMPK. Furthermore, these data are supported by the finding that metformin uptake in the brain can be mediated by the organic cation transporter 3 (OCT3), a member of the SLC22A family, which in turn modulates the pharmacologic action of metformin on AMPK [41]. After metformin chronic treatment BACE1 and APP, the enzyme-substrate required for Aβ production, are differently modulated, mainly in the cortex where an enhanced APP processing was assumed. This result coincides with the discovery that metformin increases Aβ generation in cultured neurons due to induction of BACE expression [17]. Modulation of BACE transcription has been reported to depend on the pathway involving activated AMPK, being antagonized by the AMPK inhibitor compound C [17]. It has been postulated that metformin modulates BACE1 and APP transcription activating a signal including AMPK and leading to increase Aβ production [17]. More recently it has been demonstrated that metformin induces up-regulation of APP and PSN1 through a mechanism involving oxidative stress, mitochondrial dysfunction and Nf-κB activation [21]. Further support to these findings was given by immunofluorescence analysis in the mouse brain regions that are vulnerable in AD pathogenesis. The inspection showed a large number of Aβ aggregates in the cerebral cortex, whereas no significant Aβ deposits were detected in the hippocampus. In particular, in the cortex we found a quite different distribution of APP in control and metformin treated mice, confirming that an increased processing leading to Aβ oligomerization or aggregate formation were occurred.

However, this result was in agreement with the evolution of the temporal-spatial accumulation of Aβ described in AD [42]. In line with these results, increase of typical AD biomarkers and Aβ aggregates in the cortex area and no in the hippocampus, denotes that we have focalized a molecular moment comparable to an early neurodegenerative stage. However, we can hypothesize that the different effects of metformin in cortex and hippocampus areas could be due to a diverse metformin distribution or accumulation. In fact, analysis of seven rat brain regions, by HPLC method, has demonstrated that metformin concentrations varied in the different brain regions and, both after acute and chronic administration, it was higher in the cortex than in the hippocampus [43].

Our findings suggest that increased APP and Aβ production, due to metformin exposition, could impair mitochondrial function in brain neurons acting in multiple ways on different targets. Metformin treatment changes the levels of expression of TOM40, VDAC1 and HKI, proteins involved in mitochondrial import and export of molecules and metabolites, in cortex region where Aβ aggregates are mainly present. Studies on human brain biopsies have demonstrated that TOM40 pore mediates the internalization of Aβ and APP [44]. Depending on the size of the molecules, the whole APP can block TOM40 channel and the small Aβ can be imported in the inner membrane where affects the respiratory chain. Moreover, influx of Aβ via the TOM40 pore increases Reactive Oxygen Species (ROS) within the organelle, leading to mitochondrial dysfunction and structural and functional damage of neurons [45]. In addition, ROS triggers events that include the increase of Aβ production thus nourishing a vicious circle by which Aβ self-feed its own production [21]. Thus, increased TOM40 could favor transport of Aβ into the mitochondria and their impairing. In their physiological state, the dimensions of the VDAC1 pores are sufficient to selectively allow the passage of small molecules. During cell death processes, in which the release of folded proteins like cytochrome C is required, the formation of larger channels is necessary [46]. In line with this study we found VDAC1 in monomeric, with different electrophoretic mobility, dimeric and oligomeric configurations. VDAC1 is a β-barrel protein with a flexible N-Terminal domain that can be located within the pore by intramolecular crosslinking (Cys-Lys) or exposed to the cytosolic face that could associate with the N-Terminal region of another VDAC1 molecule leading to oligomerization [47]. Moreover, using different stimuli it has been demonstrated that the lower monomeric band appears only when apoptosis is induced [48], indicating that a neurodegenerative process has been locally produced. Furthermore, VDAC1 N-terminus has been found mediate the interaction of VDAC1 with anti-apototic proteins such as HKI [47], and VDAC1 oligomerization is considered relevant for interactions with proteins involved in apoptosis [46]. In the cortex region, where metformin-induced Aβ aggregates are accumulated, and apoptotic neurons were observed, a decrease in the amount of monomers and an increase of intramolecular crosslinked products with a concomitant increase in oligomers formation were found. Furthermore, decrease of HKI suggests that its binding to VDAC1 was displaced and the increased concentration of free VDAC1 molecules has promoted VDAC1 oligo-merization. This is in line with the observation that Aβ mediated neurodegeneration involves detachment of HKI from VDAC1 that oligomerizes and promotes cytochrome C release, events leading to apoptosis [28]. Thus, the increased presence of Aβ, due to metformin treatment, could compete with the binding of HKI and/or other anti-apoptotic proteins with VDAC1 and be the apoptotic stimulus for enhancing VDAC1 oligomerization to produce enlarged pores capable to change permeability and mediate cytochrome C release, in critical brain region. In support of this observation direct interaction of Aβ and phosphorylated tau with VDAC1 has been demonstrated [27,28]. Aβ interaction is able to block mitochondrial pores and interfere with the transport of ATP, ADP and other metabolites between mitochondria and cytoplasm, leading to mitochondrial dysfunction and neurodegeneration [27]. Thus, the observed related increase of VDAC1, Aβ and apoptotic cells, due to metformin administration, mainly in the cortex, could be an initial neurodegenerative event that could be spread to other brain regions as the disease progresses. However, this concept need of additional experimental supports. Lastly, being mitochondrial dysfunction considered an early pathophysiological event in AD, TOM40 and VDAC1 may be proposed as potential timeline biomarkers for this pathology [49]. Consistent with data is the change in expression levels of TOM40 and VDAC1 mainly in cerebral cortex, in a very local specific manner. However, our observations suggest a strong association between metformin-induced Aβ accumulation and mitochondrial dysfunction via impaired TOM40 and VDAC1 expression that is cause of mitochondrial stress and, in turn, source of increased Aβ production. Because many of these factors can be either a cause or a consequence of the others, it is difficult to establish a clear sequence of events. Finally, we cannot exclude that the effect of metformin seen in brain of mice after three months of exposition could be exacerbated by the damage induced by the physiological aging. However, the potentiality of this drug to produce side effects could be counteracted, as suggested in other studies, by its use in combination with insulin [17,18,21].

Very interestingly, by biophysical methods, our study also demonstrates that metformin is able to directly interact with Aβ amyloid species involved in AD influencing their aggregation kinetics and features. Indeed, in the presence of metformin the typical sigmoidal profile of the fibrillogenesis shows an increased lag-phase and final reduction of amyloid fibrils, stabilizing the prefibrillar oligomeric species. The direct influence on lag-phase reveals that metformin is able to influence the nucleation step of the process by the formation of a drug-Aβ complex [50]. The molecule could exert a stabilizing effect on the on-pathway seeding species involved in β-sheet formation and monomer amyloid assembly.

From DLS and AFM, the presence of metformin sizably reduces the formation of large amyloid fibers, favoring the formation of smaller aggregates. Furthermore, these aggregates have dimensions comparable to that observed in mouse brains even if we cannot exclude that in vivo the size of the aggregates could be influenced by other entrapped extracellular components [3].

These results could provide the hypothesis on a different route by which, together with the others above described, metformin could be negatively correlated to AD pathogenesis. Indeed, in the wide structural poly-morphism of Aβ oligomers, the accredited assumption is that, rather than mature fibrils, the species more involved in pathogenesis of AD are prefibrillar oligomeric species that form at the beginning of the process [51,52]. These species expose larger hydrophobic surfaces to the solvent and, therefore, could directly interact with cell membranes influencing calcium homeostasis and ROS production. It cannot be excluded, therefore, that the smaller species stabilized by metformin in vitro are those with higher cytotoxic potentiality, whereas mature fibrils, considered harmless, are much reduced in length and number. In this respect, also by a direct action on the product of APP cleavage, i.e. Aβ peptide, the use of metformin could contribute to toxicity associated to AD.

**Figure 9:**
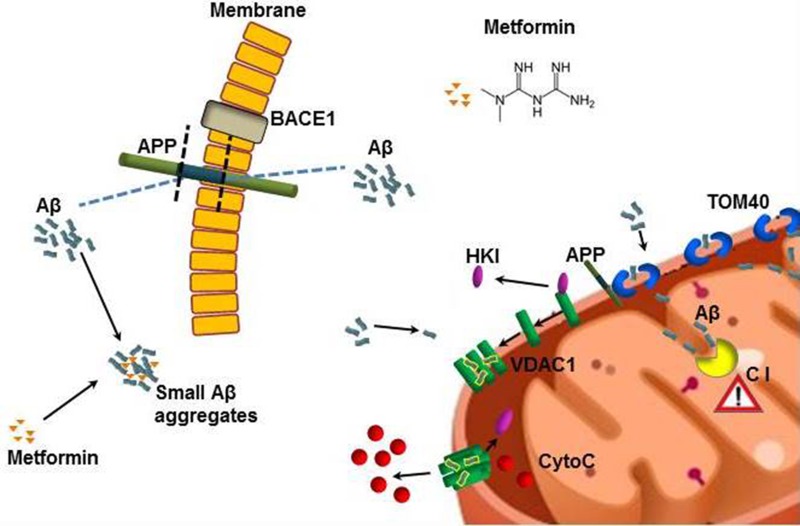
Model describing how metformin induces Aβ aggregates formation and mitochondrial dysfunction Metformin increases BACE1 production that stimulates APP processing and Aβ production at cell membrane level. Metformin stabilizes small Aβ aggregates that could be internalized. TOM40 pore mediates the mitochondrial internalization of Aβ and APP. The whole APP can block TOM40 channel and the small Aβ can be imported in the inner membrane where affects the Complex I (C I) of the respiratory chain. Small Aβ aggregates displace the binding of HKI with mitochondrial VDAC1 leading to its oligomerization and the formation of large pores that are capable to change permeability and mediate the cytochrome C release. All these coexistent events lead to mitochondrial dysfunction and neuronal apoptosis.

The high prevalence of AD and T2DM in the elderly population suggests that concomitant pharmacotherapy could be desirable. Our findings indicate that metformin, the drug usually administered for T2DM, is able to reach the brain in C57B6/J mice, where it activates neurodegenerative pathways, including mitochondrial dysfunction and apoptosis, mainly in the brain cortex. Furthermore, metformin is able to directly interacts in vitro with Aβ, modifying its aggregation profile, reducing the amount of mature fibrils and stabilizing toxic prefibrillar oligomeric species. A summary of the proposed mechanism is shown in figure 9. Thus, metformin induces different adverse effects, possibly leading to an overall increase of the risk of AD onset.

## MATERIALS AND METHODS

### Mice

The experimental procedures employed in the present study were in accordance with the Italian D.L. no. 116 of 27 January 1992 and subsequent variations and the recommendations of the European Economic Community (86/609/ECC). The studies were approved by Ministero della Sanità (Rome, Italy). Male C57BL/6J (B6) mice, purchased from Harlan Laboratories (San Pietro al Natisone Udine, Italy) at 4 weeks of age, were housed under standard conditions of light (12 h light: 12 h darkness cycle) and temperature (22–24 °C), with free access to water and food. After acclimatization (1 week), the animals were divided in two groups (n=10 per group), control and metformin treated. Mice were provided food and received metformin in drinking water (2 mg/mL) for 7 days or three months. After this treatment, animals were sacrificed by cervical dislocation and the brain of age-matched animals were immediately exported and processed for subsequent analysis, as previously described [7]. When necessary, hippocampus and cortex were separated. Biodistribution of metformin in ex vivo brain was detected by using Biospace Lab Imaging Instrument.

### Total protein extraction and western blotting

Brain of mice were homogenized in RIPA buffer (20 mM Tris, pH 7.4, 150 mM NaCl, 1 mM Na3VO4, 10 mM NaF, 1mM EDTA, 1 mM EGTA, 0.2 mM phenyl-methylsulfonyl fluoride, 1% Triton, 0.1%SDS, and 0.5% deoxycholate) with protease inhibitors (Amersham) and phosphatase inhibitor cocktail II and III (SIGMA). To remove insoluble material, tissue lysates were sonicated and centrifuged (14,000 rpm, at 4°C, for 30 min). Proteins (50 μg) were resolved by 10% SDS-PAGE gel and transferred onto nitrocellulose filters for Western blotting using anti-APP (1:1000), anti-BACE1 (1:1000) anti-phospho-AMPK (1:1000), anti-TOM40 (1:1000), anti-VDAC1 (1:1000), and anti-HKI (1:1000) purchased from Santa Cruz, anti-β-actin (1:5000) purchased from Sigma. Secondary antibodies conjugated to horseradish peroxidase (1:2000) purchased from Cell Signaling were detected using the NOVEX® ECL HRP chemiluminescence kit (Cat. n° WP20005, Invitrogen) according to the manufacturer's instructions. In some instances, antibodies were stripped from blots with Restore Western Blot Stripping Buffer (Thermo Scientific) for 10 minutes at room temperature, for antibody reprobing. Band intensities were analyzed with a gel documentation system (BioRad), expression was normalized with β-actin expression. The protein levels were expressed as densitometry and percentage of controls.

### Immunofluorescence

For immunofluorescence the brains (n=5 per group) were embedded in paraffin as previously described [7] and coronally sectioned (5μm) using a microtome. Brain sections including the cerebral cortex and the hippocampus were mounted on slides and deparaffinized in xylene solution. Then, the slides were hydrated in a series of graded ethanol (96%, 85%, 70%, 50%) for 5 minutes each. After washing in water and PBS the slides were incubated with 3% BSA/PBS for 1 h. Next, the sections were incubated with anti-APP (1:50) (Santa Cruz) at 4°C overnight. After washing in PBS, the samples were incubated with anti-rabbit Cy3-conjugate secondary antibody (1:500; SIGMA). For nuclear staining, the sections were incubated with Hoechst 33258 (5μg/ml) for 20 minutes. After washing in PBS the slides were mounted with cover slips and images were visualized by using a Leica DM5000 upright microscope (Leica Microsystems, Heidelberg, Germany) at 20X magnification.

### Thioflavin T staining

For Thioflavin-T (ThT) staining the brain sections including the cerebral cortex and the hippocampus were mounted on slides The slides were deparaffinized in xylene solution and hydrated in a series of graded ethanol (96%, 85%, 70%, 50%) for 5 minutes each. After washing in water, the sections were incubated in filtered 1% aqueous ThT solution for 8 minutes at room temperature. The slides were then dehydrated in ethanol 80% and 95%, for 5 minutes each. After washing in water the slides were mounted with cover slips and the images were visualized by using a Leica DM5000 upright microscope (Leica Microsystems, Heidelberg, Germany) at 20x magnification.

### TUNEL assay

Terminal deoxynucleotidyl Transferase Biotin-dUTP Nick End Labeling (TUNEL)-positive apoptotic nuclei were detected in brain paraffin sections using an in situ cell death detection kit (Promega) according to manufacturer's instructions. The number of apoptotic cells was counted in randomly selected fields to calculate the ratio of apoptotic cell per brain area.

### Quantitative real-time PCR

Total RNA was extracted using RNEasy Mini Kit (Qiagen). Two ng of RNA was used to synthesize the first strand cDNA using RT First-Strand kit (Qiagen). Synthesized cDNAs were amplified using RT2 SYBR Green/ROX qPCR Mastermix (Qiagen) and StepOne Real-Time instrument (Applied Biosystem). Gene expression validation was performed using RT2 qPCR Primer Assay for human APP, Presenilin1, β-actin (SABiosciences). Gene expression was normalized to β-actin.

### Statistical analysis

All experiments were repeated at least three times and each experiment was performed in triplicate. The results are presented as mean + SD. A one-way ANOVA was performed, followed by Dunnet's post hoc test for analysis of significance. Results with a p-value <0.05 were considered statistically significant, *P < 0.05, **P<0.02.

### Sample preparation for biophysics experiments

The synthetic peptide Aβ_1–40_ (Anaspec) was pretreated according to the procedure of Fezoui et al. [53] for improving the reliability of experiments at neutral pH. Stock aliquots (200 μg each) were stored at −80°C. Metformin was purchased from Sigma Aldrich. Aβ_1–40_ samples were prepared by dissolving the lyophilized peptide in 50 mM phosphate buffer, pH 7.4, at a concentration of about 70 μM. The solution was filtered through 0.22 μm and 20 nm filters into a fluorescence quartz cuvette containing a small magnetic stirring bar. Aβ_1–40_ concentration was determined by tyrosine absorption at 276 nm using an extinction coefficient of 1390 cm^−1M−1^. The sample was then diluted to the working concentration of 50 μ M by adding the appropriate amount of buffer, concentrated solution of ThT (1mM), and concentrated solution of metformin (20 mM) when required.

Final samples containing Aβ and metformin were obtained by appropriate aseptically mixing of the protein solutions and placed in closed cuvettes in a cold room at 4°C, before incubation at higher temperatures. The aggregation kinetics were followed at controlled temperature (37°C) and under controlled stirring (200 rpm) for 24 hours.

### ThT spectrofluorometric measurements

ThT fluo-rescence emission was monitored by using a JASCO FP-6500 spectrometer. The excitation and emission wavelengths were 450 and 485 nm, respectively, with 3 nm slit width. ThT concentration was 12 μM. The sample was placed at 37°C in the thermostated cell compartment (10 mm). When required, a magnetic stirrer at 200 rpm (mod. 300, Rank Brothers Ltd., Cambridge) was used.

Control fluorimetric experiments between Thioflavin T and metformin with the purpose of excluding undesired pitfalls due to potential interaction between the dye and the molecule [54] have been performed under the same conditions of aggregation kinetics (37°C, under stirring).

### Circular dichroism spectroscopy

CD measurements were acquired by using a JASCO J-815 CD Spectrometer. Particularly, during the aggregation kinetics, withdrawals of samples at appropriate time were observed. Spectra were recorded at 20°C using a quartz cell with 0.2 mm path length. Each spectrum measurement was obtained by averaging over eight scans and subtracting the blank solvent contribution.

The aggregation of Aβ peptide in the presence or absence of metformin was investigated by Dynamic Light Scattering. The samples were placed into a dust-free quartz cell without further filtering and kept at 37°C in the thermostatic cell compartment of a Brookhaven Instruments BI200-SM goniometer. The temperature was controlled within 0.1°C using a thermostatic recirculating bath. The light scattered intensity and its autocorrelation function were measured at θ = 90° by using a Brookhaven BI-9000 correlator and a 50 mW He–Ne laser tuned at a wavelength λ = 632.8 nm. Due to their Brownian motion, particles moving in solution give rise to fluctuations in the intensity of the scattered light [55, 56]. The autocorrelator measures the homodyne intensity–intensity correlation function that, for a Gaussian distribution of the intensity profile of the scattered light, is related to the electric field correlation function:

$$g^{\left(2\right)}(q,t)={\left[A+Bg^{\left(1\right)}(q,t)\right]}^{2}$$ (1)

where *A* and *B* are the experimental baseline and the optical constant, respectively. For polydisperse particles*, g^(1)^(q,t)* is given by:

$$g^{\left(1\right)}(q,t)=\int_{0}^{\infty }G(\Gamma )\exp(-\Gamma t)d\Gamma$$ (2)

Here, *G*(Γ) is the normalized number distribution function for the decay constant *Γ = q^2^D_T_*, where *q=(4πn/λ)sin(θ/2)* is the scattering vector defining the spatial resolution with *n* and *D_T_* being the solvent refractive index and the translational diffusion coefficient, respectively. The hydrodynamic diameter *D_H_* is calculated from *D_T_* through the Stokes–Einstein relationship:

$$D_{T}=\frac{k_{B}T}{3\pi \eta D_{H}}$$ (3)

where *k_B_* is the Boltzmann constant, *T* is the absolute temperature, and *η* is the solvent viscosity. Intensity-weighted distribution functions P_I_ of the z-average hydrodynamic diameter D_H_ were obtained by the analysis of the intensity autocorrelation functions were analyzed by means of a CONTIN-like smoothing-constrained regularization method [57].

### Atomic Force Microscopy (AFM)

AFM measurements were performed by using a Nanowizard III (JPK Instruments, Germany) mounted on an Axio Observer D1 (Carl Zeiss, Germany) or on an Eclips Ti (Nikon, Japan) inverted optical microscope. Aliquots of protein solutions were deposited onto freshly cleaved mica surfaces (Agar Scientific, Assing, Italy) and incubated for up to 20 min before rinsing with deionized water and drying under a low pressure nitrogen flow. Imaging of the protein was carried out in intermittent contact mode in air by using NCHR silicon cantilever (Nanoworld, Switzerland) with nominal spring constant ranging from 21 to 78 N/m and typical resonance frequency ranging from 250 to 390 kHz.

## References

[1] Ott A, Stolk RP, van Harskamp F, Pols HA, Hofman A, Breteler MM (1999). Diabetes mellitus and the risk of dementia: the Rotterdam Study. Neurology.

[2] Alzheimer's Association (2015). Alzheimer's disease facts and figures. Alzheimer's & Dementia.

[3] Selkoe DJ (1998). The cell biology of β-amyloid precursor protein and presenilin in Alzheimer's disease. Trends Cell Biol.

[4] Walsh DM, Klyubin I, Fadeeva JV, Cullen WK, Anwyl R, Wolfe MS, Rowan MJ, Selkoe DJ (2002). Naturally secreted oligomers of amyloid beta protein potently inhibit hippocampal long-term potentiation in vivo. Nature.

[5] Weingarten MD, Lockwood AH, Hwo SY, Kirschner MW (1975). A protein factor essential for microtubule assembly. Proc Natl Acad Sci USA.

[6] Hildreth KL, Van Pelt RE, Schwartz RS (2012). Obesity, insulin resistance, and Alzheimer's disease. Obesity (Silver Spring).

[7] Nuzzo D, Picone P, Baldassano S, Caruana L, Messina E, Marino Gammazza A, Cappello F, Mulè F, Di Carlo M (2015). Insulin resistance as common molecular denominator linking obesity to Alzheimer's disease. Curr Alzheimer Res.

[8] Watson GS, Craft S (2003). The role of insulin resistance in the pathogenesis of Alzheimer's disease: implications for treatment. CNS Drugs.

[9] Watson GS, Craft S (2004). Modulation of memory by insulin and glucose: neuropsychological observations in Alzheimer's disease. Eur J Pharmacol.

[10] De Felice FG (2013). Alzheimer's disease and insulin resistance: translating basic science into clinical applications. J Clin Invest.

[11] Ghasemi R, Haeri A, Dargahi L, Mohamed Z, Ahmadiani A (2013). Insulin in the brain: sources, localization and functions. Mol Neurobiol.

[12] Picone P, Giacomazza D, Vetri V, Carrotta R, Militello V, San Biagio PL, Di Carlo M (2011). Insulin-activated Akt rescues Aβ oxidative stress-induced cell death by orchestrating molecular trafficking. Aging Cell.

[13] Steen E, Terry BM, Rivera EJ, Cannon JL, Neely TR, Tavares R, Xu XJ, Wands JR, de la Monte SM (2005). Impaired insulin and insulin-like growth factor expression and signaling mechanisms in Alzheimer's disease--is this type 3 diabetes?. J Alzheimers Dis.

[14] Di Carlo M, Picone P, Carrotta R, Giacomazza D, San Biagio PL (2010). Insulin promotes survival of amyloid-beta oligomers neuroblastoma damaged cells via caspase 9 inhibition and Hsp70 upregulation. J Biomed Biotechnol.

[15] Picone P, Ditta LA, Sabatino MA, Militello V, San Biagio PL, Di Giacinto ML, Cristaldi L, Nuzzo D, Dispenza C, Giacomazza D, Di Carlo M (2016). Ionizing radiation-engineered nanogels as insulin nanocarriers for the development of a new strategy for the treatment of Alzheimer's disease. Biomaterials.

[16] Wang J, Gallagher D, DeVito LM, Cancino GI, Tsui D, He L, Keller GM, Frankland PW, Kaplan DR, Miller FD (2012). Metformin activates an atypical PKC-CBP pathway to promote neurogenesis and enhance spatial memory formation. Cell Stem Cell.

[17] Chen Y, Zhou K, Wang R, Liu Y, Kwak YD, Ma T, Thompson RC, Zhao Y, Smith L, Gasparini L, Luo Z, Xu H, Liao FF (2009). Antidiabetic drug metformin (GlucophageR) increases biogenesis of Alzheimer's amyloid peptides via up-regulating BACE1 transcription. Proc Natl Acad Sci USA.

[18] Beeri MS, Schmeidler J, Silverman JM, Gandy S, Wysocki M, Hannigan CM, Purohit DP, Lesser G, Grossman HT, Haroutunian V (2008). Insulin in combination with other diabetes medication is associated with less Alzheimer neuropathology. Neurology.

[19] Imfeld P, Bodmer M, Jick SS, Meier CR (2012). Metformin, other antidiabetic drugs, and risk of Alzheimer's disease: a population-based case-control study. J Am Geriatr Soc.

[20] Zhou G, Myers R, Li Y, Chen Y, Shen X, Fenyk-Melody J, Wu M, Ventre J, Doebber T, Fujii N, Musi N, Hirshman MF, Goodyear LJ, Moller DE (2001). Role of AMP-activated protein kinase in mechanism of metformin action. J Clin Invest.

[21] Picone P, Nuzzo D, Caruana L, Messina E, Barera A, Vasto S, Di Carlo M (2015). Metformin increases APP expression and processing via oxidative stress, mitochondrial dysfunction and NF-κB activation: use of insulin to attenuate metformin's effect. Biochim Biophys Acta.

[22] Schäfer G, Rieger E (1974). Interaction of biguanides with mitochondrial and synthetic membranes. Effects on ion conductance of mitochondrial membranes and electrical properties of phospholipid bilayers. Eur J Biochem.

[23] Detaille D, Guigas B, Leverve X, Wiernsperger N, Devos P (2002). Obligatory role of membrane events in the regulatory effect of metformin on the respiratory chain function. Biochem Pharmacol.

[24] Carvalho C, Correia S, Santos MS, Seiça R, Oliveira CR, Moreira PI (2008). Metformin promotes isolated rat liver mitochondria impairment. Mol Cell Biochem.

[25] Suski M, Olszanecki R, Chmura Ł, Stachowicz A, Madej J, Okoń K, Adamek D, Korbut R (2016). Influence of metformin on mitochondrial subproteome in the brain of apoE knockout mice. Eur J Pharmacol.

[26] Gottschalk WK, Lutz MW, He YT, Saunders AM, Burns DK, Roses AD, Chiba-Falek O (2014). The broad impact of TOM40 on neurodegenerative diseases in aging. J Parkinsons Dis Alzheimers Dis.

[27] Manczak M, Reddy PH (2012). Abnormal interaction of VDAC1 with amyloid beta and phosphorylated tau causes mitochondrial dysfunction in Alzheimer's disease. Hum Mol Genet.

[28] Smilansky A, Dangoor L, Nakdimon I, Ben-Hail D, Mizrachi D, Shoshan-Barmatz V (2015). The voltage-dependent anion channel 1 mediates amyloid β toxicity and represents a potential target for Alzheimer disease therapy. J Biol Chem.

[29] Karran E, Mercken M, De Strooper B (2011). The amyloid cascade hypothesis for Alzheimer's disease: an appraisal for the development of therapeutics. Nat Rev Drug Discov.

[30] Carrotta R, Di Carlo M, Manno M, Montana G, Picone P, Romancino D, San Biagio PL (2006). Toxicity of recombinant beta-amyloid prefibrillar oligomers on the morphogenesis of the sea urchin Paracentrotus lividus. FASEB J.

[31] Picone P, Carrotta R, Montana G, Nobile MR, San Biagio PL, Di Carlo M (2009). Abeta oligomers and fibrillar aggregates induce different apoptotic pathways in LAN5 neuroblastoma cell cultures. Biophys J.

[32] Mannini B, Mulvihill E, Sgromo C, Cascella R, Khodarahmi R, Ramazzotti M, Dobson CM, Cecchi C, Chiti F (2014). Toxicity of protein oligomers is rationalized by a function combining size and surface hydrophobicity. ACS Chem Biol.

[33] Novitskaya V, Bocharova OV, Bronstein I, Baskakov (2006). Amyloid fibrils of mammalian prion protein are highly toxic to cultured cells and primary neurons. J Biol Chem.

[34] Bucciantini M, Nosi D, Forzan M, Russo E, Calamai M, Pieri L, Formigli L, Quercioli F, Soria S, Pavone F, Savistchenko J, Melki R, Stefani M (2012). Toxic effects of amyloid fibrils on cell membranes: the importance of ganglioside GM1. FASEB J.

[35] Bucciantini M, Rigacci S, Stefani M (2014). Amyloid aggregation: role of biological membranes and the aggregate-membrane system. J Phys Chem Lett.

[36] Lomakin A, Teplow DB, Kirschner DA, Benedek GB (1997). Kinetic theory of fibrillogenesis of amyloid beta-protein. Proc Natl Acad Sci USA.

[37] Lee CC, Nayak A, Sethuraman A, Belfort G, McRae GJ (2007). A three-stage kinetic model of amyloid fibrillation. Biophys J.

[38] LeVine H, Thioflavine T (1993). interaction with synthetic Alzheimer's disease beta-amyloid peptides: detection of amyloid aggregation in solution. Protein Sci.

[39] Naiki H, Higuchi K, Hosokawa M, Takeda T (1989). Fluorometric determination of amyloid fibrils in vitro using the fluorescent dye, thioflavin T1. Anal Biochem.

[40] Coelho-Cerqueira E, Pinheiro AS, Follmer C (2014). Pitfalls associated with the use of Thioflavin-T to monitor anti-fibrillogenic activity. Bioorg Med Chem Lett.

[41] Chen L, Pawlikowski B, Schlessinger A, More SS, Stryke D, Johns SJ, Portman MA, Chen E, Ferrin TE, Sali A, Giacomini KM (2010). Role of organic cation transporter 3 (SLC22A3) and its missense variants in the pharmacologic action of metformin. Pharmacogenet Genomics.

[42] Khan UA, Liu L, Provenzano FA, Berman DE, Profaci CP, Sloan R, Mayeux R, Duff KE, Small SA (2014). Molecular drivers and cortical spread of lateral entorhinal cortex dysfunction in preclinical Alzheimer's disease. Nat Neurosci.

[43] Łabuzek K, Suchy D, Gabryel B, Bielecka A, Liber S, Okopień B (2010). Quantification of metformin by the HPLC method in brain regions, cerebrospinal fluid and plasma of rats treated with lipopolysaccharide. Pharmacol Rep.

[44] Hansson Petersen CA, Alikhani N, Behbahani H, Wiehager B, Pavlov PF, Alafuzoff I, Leinonen V, Ito A, Winblad B, Glaser E, Ankarcrona M (2008). The amyloid β-peptide is imported into mitochondria via the TOM import machinery and localized to mitochondrial cristae. Proc Natl Acad Sci USA.

[45] Reddy PH (2009). Amyloid beta, mitochondrial structural and functional dynamics in Alzheimer's disease. Exp Neurol.

[46] Zalk R, Israelson A, Garty ES, Azoulay-Zohar H, Shoshan-Barmatz V (2005). Oligomeric states of the voltage-dependent anion channel and cytochrome c release from mitochondria. Biochem J.

[47] Geula S, Ben-Hail D, Shoshan-Barmatz V (2012). Structure-based analysis of VDAC1: n-terminus location, translocation, channel gating and association with anti-apoptotic proteins. Biochem J.

[48] Keinan N, Tyomkin D, Shoshan-Barmatz V (2010). Oligomerization of the mitochondrial protein voltage-dependent anion channel is coupled to the induction of apoptosis. Mol Cell Biol.

[49] Reddy PH (2013). Is the mitochondrial outer membrane protein VDAC1 therapeutic target for Alzheimer's disease?. Biochim Biophys Acta.

[50] Bartolini M, Bertucci C, Bolognesi ML, Cavalli A, Melchiorre C, Andrisano V (2007). Insight into the kinetic of amyloid beta (1-42) peptide self-aggregation: elucidation of inhibitors' mechanism of action. ChemBioChem.

[51] Hata S, Saito Y, Suzuki T, Jelinek R (2011). Alzheimer's Disease as a Membrane-Associated Enzymopathy of Δ-Amyloid Precursor Protein (APP) Secretases. Lipids and cellular membranes in amyloid diseases.

[52] Canale C, Seghezza S, Vilasi S, Carrotta R, Bulone D, Diaspro A, San Biagio PL, Dante S (2013). Different effects of Alzheimer's peptide Aβ(1-40) oligomers and fibrils on supported lipid membranes. Biophys Chem.

[53] Fezoui Y, Hartley DM, Harper JD, Khurana R, Walsh DM, Condron MM, Selkoe DJ, Lansbury PT, Fink AL, Teplow DB (2000). An improved method of preparing the amyloid beta-protein for fibrillogenesis and neurotoxicity experiments. Amyloid.

[54] Hudson SA, Ecroyd H, Kee TW, Carver JA (2009). The thioflavin T fluorescence assay for amyloid fibril detection can be biased by the presence of exogenous compounds. FEBS J.

[55] Pusey PN, Zemb Th, Lindner P (2002). Introduction to scattering experiments. Netrons, X-ray and light: scattering method applied to soft condensed matter.

[56] Berne BJ, Pecora R (1976). Dynamic Light Scattering.

[57] Stepanek P, Brown W (1993). The method and some applications. Dynamic ligth scattering.

